# Intratumoral temozolomide in spontaneous canine gliomas: feasibility of a novel therapy using implanted microcylinders

**DOI:** 10.1002/vms3.124

**Published:** 2018-11-05

**Authors:** Jill Hicks, Simon Platt, Georgina Stewart, Christine Senneca, Shannon Holmes, Marc Kent, Elizabeth Howerth, Jared Kaplan, Edward Kaplan

**Affiliations:** ^1^ Veterinary Teaching Hospital University of Georgia Athens Georgia USA; ^2^ Small Animal Hospital University of Florida Gainesville FL USA; ^3^ Department of Internal Medicine Yale Medical School New Haven CT USA; ^4^ Microspherix LLC Bocan Raton FL USA; ^5^ Present address: Blue Pearl Veterinary Partners Grand Rapids MI US; ^6^ Present address: AXIS ‐ Animal Cross‐Sectional Imaging Specialists Athens GA USA

**Keywords:** entotherapy, glioma, chemotherapy, magnetic resonance imaging, canine

## Abstract

Entotherapy[Link] an image‐guided drug‐eluting microcylinder platform, has the potential to bypass the limitations of systemic chemotherapy use in the treatment of canine brain tumours. Gliomas, which are common in dogs and also represent the majority of fatal brain tumours in humans, can be amenable to chemotherapy with temozolomide. Biopolymer microcylinders conjugated with temozolomide and gadolinium were implanted into partially resected tumours of four client‐owned dogs with gliomas. All four dogs presented with generalized seizures and had mild to no neurologic deficits at the time of craniotomy. All dogs underwent craniotomy for implantation of the microcylinders into partially resected gliomas (glioblastoma multiforme {*n* = 1} or oligodendroglioma {*n* = 3}). All dogs recovered well from the craniotomy and implantation procedure. This novel procedure appears to be feasible and tolerated in tumour‐bearing dogs. A future controlled clinical study can now aim to evaluate the microcylinder implantation for long‐term efficacy.

## Introduction

Gliomas, tumours of the neuroectoderm, are one of the two most common primary brain tumours in dogs along with meningiomas (Song *et al*. 2013), and are believed to carry a poor long‐term prognosis, though it has been difficult to gauge outcome in much of the published data. Median survival times of patients with presumptive intracranial gliomas are documented to be 7.6 to 14 months following radiation therapy with or without adjunctive temozolomide (Hu *et al*. 2015; Dolera *et al*. 2017), 69 days with symptomatic care (Rossmeisl *et al*. 2013) and around 6 months, likely related to grade, following surgical resection with adjuvant therapy (MacLellan *et al*. 2018). In humans, gliomas typically carry a guarded prognosis as well, with high‐grade glioma having 1 and 5‐year survival times at 40% and <10%, respectively (Louis *et al*. 2016). Grade I and II gliomas (low grade) in humans carry a more favourable prognosis overall, but are still a uniformly fatal disease with a median survival time of 7 years (Lote *et al*. 1997) and nearly half of low grade gliomas undergoing high grade transformation within about two years following diagnosis (Claus *et al*. 2015). Given that gliomas are such an important disease of both dogs and humans, it is logical that dogs have become an important spontaneous model for brain tumour therapy (Hicks *et al*. 2015), with significant interest being shown across the entirety of veterinary neuro‐oncology and the National Cancer Institute (Leblanc *et al*. 2016). Fortunately, this mutually beneficial relationship allows us to advance research for human glioma treatment while providing resources to improve the poor long‐term prognosis that gliomas convey in our canine patients.

Among the strategies developed for drug delivery into the CNS, locally controlled drug release by the way of an implantable polymeric device has been developed in recent years (Menei *et al*. 1996, 2005a; Benoit *et al*. 2000; Chen *et al*. 2005; Manome *et al*. 2006; Yemisci *et al*. 2006; Suchorska *et al*. 2011). The first polymeric devices developed were macroscopic implants requiring open surgery for implantation. Over the last few years, poly(lactide‐co‐glycolide) {PLGA} microcylinders have been shown to be safe and promising for drug delivery into the brain (Benoit *et al*. 2000; Menei *et al*. 2005a). PLGA is biodegradable and biocompatible with brain tissue. Due to their size, these microcylinders can be easily implanted by stereotaxy in discrete, precise and functional areas of the brain without causing damage to the surrounding‐tissue (Benoit *et al*. 2000; Menei *et al*. 2005a). Brain tumour treatments have been developed using this approach and clinical trials have been performed (Menei *et al*. 1996; Chen *et al*. 2005; Manome *et al*. 2006; Yemisci *et al*. 2006; Suchorska *et al*. 2011). In one such study, biodegradable microcylinders loaded with the radiosensitizer 5‐fluorouracil were successfully implanted by stereotaxy into inoperable gliomas (Menei *et al*. 1996). Idoxuridine, ganciclovir, BCNU, 125‐iodine (125I) and doxorubicin have all been successfully conjugated with PLGA microcylinders (Chen *et al*. 2005; Manome *et al*. 2006; Yemisci *et al*. 2006; Suchorska *et al*. 2011). Stereotactic brachytherapy (SBT) with 125I microspheres has been shown to enable long, protracted, focused irradiation of brain tumours at low doses (Suchorska *et al*. 2011). It has been shown in a small number of institutional case series to be a valuable tool for first‐line treatment of small and circumscribed low grade gliomas in humans that are well delineated (according to computed tomography [CT] and/or magnetic resonance imaging [MRI] criteria) but not accessible for tumour resection due to their location (Suchorska *et al*. 2011).

Temozolomide (TMZ) chemotherapy has been the most promising adjunctive treatment in the past few decades for humans with glioblastoma multiforme, the most aggressive of all glial tumours; the 2‐year survival time increased from 10% to 27% following addition of the drug to multimodal therapy (Stupp *et al*. 2005). However, even though oral TMZ is able to cross the blood brain barrier, cerebrospinal fluid (CSF) concentrations only reach approximately 20% of plasma concentrations in both humans and rat models (Ostermann *et al*. 2004; Zhou *et al*. 2007). Intratumoral delivery of TMZ is an area of interest to simultaneously increase the cytotoxic potential and decrease systemic side effects of the drug. This concept has been supported by evidence in murine models that intratumoral delivery of TMZ is significantly more effective in improving survival time versus systemic temozolomide use, while avoiding side effects, such as transient leukopenia, which can hamper the drug's benefit (Fritzell *et al*. 2013).

Based on the concept of intratumoral delivery of TMZ, we developed a biocompatible PLGA microcylinder infused with TMZ and gadolinium (Gd) to allow good visibility of implanted microcylinders via magnetic resonance imaging (MRI). Freehand brain implantation of the TMZ/Gd microcylinders and 30 days of intraparenchymal microcylinder degradation was well tolerated in normal canines (Hicks *et al*. 2016) indicating that a pilot study could be attempted in tumour‐bearing canine patients. The aim of this preliminary study was to assess the clinical safety of TMZ conjugated PLGA microcylinder implantation into brain tumour tissue of client owned dogs with naturally occurring intracranial gliomas. Additionally, we wanted to use this opportunity to evaluate whether freehand implantation of the microcylinders into a resected tumour bed could achieve adequate distribution throughout tumour tissue based on post‐operative MRI. Although efficacy of the treatment was not a pre‐determined aim of this small trial, we wanted to assess whether MRI‐based calculations of tumour volume immediately post‐implantation were different to that measured on a 1‐month post‐implantation MRI. The technique of implantation of TMZ/Gd microcylinders, immediate post‐surgical outcome, short‐term outcome and tolerability are described in four dogs with intracranial gliomas.

## Materials and methods

### Patient selection

Dogs were included in this clinical trial following either (1) an MRI of the brain showing a space‐occupying lesion suspected to be a glioma based on its intra‐axial mass‐like appearance and other typical imaging features (Cervera *et al*. 2011; Ródenas *et al*. 2011; Bentley *et al*. 2013) (dogs 2‐4) or (2) a surgical biopsy indicating a glioma (dog 1). Tumours had to be in a rostrotentorial location that would be accessible for partial resection and freehand placement of the microcylinders; patients also had to be deemed stable for anaesthesia and craniotomy by their primary clinician.

### Study design

Dog owners were presented with the process and purpose of the proposed experimental procedure and the expectations for follow‐up. Full informed client consent was obtained for all procedures. This clinical trial was approved by the University of Georgia's Institutional Animal Care and Use Committee (IACUC). All dogs underwent general anaesthesia for a pre‐implantation MRI, craniectomy (*n* = 4) with subtotal tumour resection (*n* = 3) and placement of a variable number of poly lactic‐co‐glycolic acid microcylinders labeled with Gd, and conjugated with TMZ; the formulation of the conjugated microcylinders was as previously described in our pre‐clinical study (Hicks *et al*. 2016) other than an alteration of the lactic:glycolic acid ratio in the microcylinders to allow a 90 days degradation. An over‐estimated pre‐surgical tumour volume was calculated by using the maximum length, width and height of the mass on post‐contrast T1W imaging in all planes. Only in dog 1 was the original tumour size not considered in order to estimate microcylinder number for implantation. Maximum microcylinder number was calculated to allow at least one microcylinder per 1 cm^3^ tumour pre‐resection, with consideration given to shape of tumour margins. The actual number of implanted microcylinders was based on the extent of resection during surgery and resulting apparent residual tumour. This was a modification of the stereotactic implantation protocol to allow flexibility for partially resected tumours.

Post‐operative MRI immediately followed. A minimum of 72 h of post‐surgical hospitalization was required before discharge, and follow‐up MRI under general anaesthesia was performed at 1, 3, 6 and 9 months post‐implantation or until the radiologist recorded a definitive increase in tumour size, though results here are only reported to the first post‐operative MRI. Adequate tumour coverage was defined by clearly visible inter‐microcylinder spacing of <1 cm and microcylinder presence detectable from the centre to within 0.5 cm of the periphery of the tumour based on MRI. A dose of 1 microcylinder per cm^3^ was anticipated to result in a TMZ dose previously documented to result in glioma cell death *in vitro* (Yushkevich *et al*. 2006). Pre‐anaesthetic screening included a complete blood count, serum chemistry, urinalysis (when possible) and thoracic radiographs within 30 days of craniectomy. Abdominal ultrasound was performed if there was a clinical indication based on blood‐work, history or physical examination.

### MRI pre and post‐implantation

Pre‐operative brain MRI was performed on a Siemens Skyra (Malverne, PA, USA) 3.0T magnet for all patients to allow planning for microcylinder implantation. All dogs had at least T2‐weighted (T2W) or T2W fluid attenuated inversion recovery (FLAIR) sequences in the transverse plane and post‐contrast T1‐weighted (T1W) magnetization prepared rapid acquisition gradient echo (MP‐RAGE) in all three planes.

Post‐craniectomy and implantation imaging were performed in all dogs. All dogs had at least T2W FLAIR sequences in the transverse plane. Dogs 1, 3 and 4 had T2W 3‐D true fast imaging with steady state free procession (TRUFI) images. Dogs 2 and 4 had post‐contrast T1W MP‐RAGE sequences. All pre‐ and post‐implantation MRIs were performed under general anaesthesia with a protocol that was determined by a board‐certified veterinary anaesthesiologist; all protocols included maintenance on isoflurane gas anaesthesia during the MRI procedure itself.

Volumetric assessment of gliomas was conducted using the open‐source ITK‐SNAP software (University of Pennsylvania, www.itksnap.org). All available MRI images for each patient were co‐registered, including T1‐ and T2‐weighted sequences. Semi‐automatic segmentation was conducted by training the program to differentiate enhancing and non‐enhancing tumour, necrosis, edema, CSF and background signal. Then active contour segmentation was automatically performed by the program to generate tumour volumes as well as three‐dimensional reconstructions of the gliomas (Yushkevich *et al*. 2006).

### Craniectomy and implantation procedure

**Figure 1:**
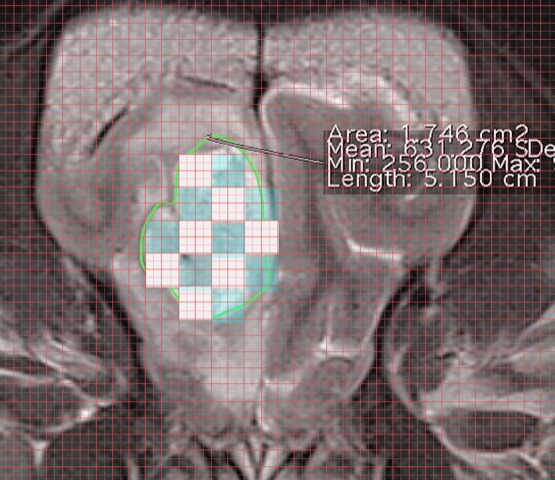
Microcylinder placement grid example.

**Figure 2:**
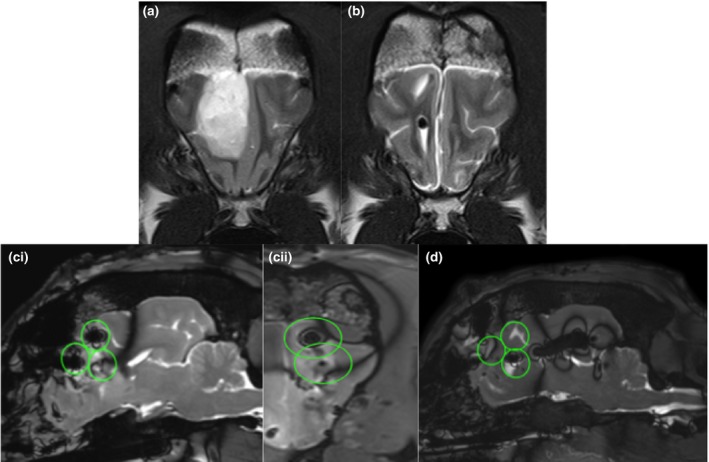
Pre‐op MRI T2W transverse image (a), 30 day post‐op T2W transverse images (b), TRUFI immediate post‐op sagittal (ci), reconstructed transverse (cii) and TRUFI sagittal plane 30 post‐op (d). A heterogeneously hyperintense distinctly marginated mass is seen in the frontal lobe prior to surgery. The tumour volume was 9.59 cm^3^. At the 30 days recheck MR examination, no tumour regrowth is seen and there is mild to moderate hemispheric atrophy. The signal void seen in the 30‐day images represent air within the ventricles. The TMZ microcylinders were best distinguished on the TRUFI images. In the immediate post‐op TRUFI images, the TMZ microcylinders are seen as focal dipoles. The green circles estimate the extent of drug dispersion, using the predicted 1 cm^3^. None of the TMZ seeds are seen 30 days post‐op depicted as empty green circles (2/3) with the 3rd circle having a void associated with gas.

All dogs underwent craniectomy for subtotal resection and tumour implantation with the TMZ/Gd microcylinders (Table 2). Dog 1, who had previously undergone a bilateral transfrontal craniotomy, was re‐approached through the same site. Dogs 2 and 4 had right rostrotentorial craniectomies. Dog 3 had a bilateral transfrontal craniotomy. TMZ/Gd microcylinder implantation was performed freehand based on measurements made from pre‐operative MRI. The goal of implantation for all dogs was to place one microcylinder per 1 cm^3^ of residual tumour tissue, with ≤0.5 cm of tumour tissue at the peripheral boundaries of the microcylinder edge (Figure 1). Each TMZ/Gd microcylinder measured 5 × 0.5 mm, contained 1 mg of TMZ and 0.25 mg gadolinium and was implanted using a brachytherapy needle. Each needle was marked at 5 mm increments to allow depth measurement. Trajectory of placement was approximated based on local skull anatomy (Figure 2). Dog 1 had three microcylinders placed into the region of previous tumour resection. A small tissue biopsy was taken in the region prior to implantation. Dogs 2, 3 and 4 had as much tumour resection as possible without increasing the likelihood of significant morbidity prior to implantation with 12, 6 and 5 microcylinders, respectively. There was a routine closure of the transfrontal craniotomy, including placement of porcine intestinal submucosa (Acell, Inc, Columbia, MD, USA) (Dog 1) over the brain, packing of the frontal sinuses with gelfoam (Pfizer, NY, NY, USA) and the replacement of the bone flap. Routine closure of the craniectomy sites did not include replacement of a bone flap or other material to cover the bony defect as there was a large amount of muscle present for protection of the region.

All dogs had post‐implantation MRI performed immediately following surgery.

### Histopathology

Brain biopsies and postmortem brain were fixed in 10% buffered formalin, routinely processed, embedded in paraffin and four unfixed sections were stained with hematoxylin and eosin. In addition, sections were stained by immunohistochemistry for oligodendrocyte transcription factor 2 (Olig 2) and glial fibrillary acidic protein (GFAP). For immunohistochemistry, heat‐induced epitope retrieval with a citrate buffer (Biogenex, San Ramon, CA) was used for both Olig2 and GFAP antibodies and the primary antibodies were rabbit anti‐Olig2 (1:400;Genetex, Irvine, CA) and mouse anti‐GFAP (1:4000;Biogenex). Tumours were considered to be oligodendrogliomas if the microscopic morphology was compatible, and the tumour cells were Olig2 positive and GFAP negative by immunohistochemistry. Grading was done using the 2016 World Health Organization Classification of Tumors of the Central Nervous System (Louis *et al*. 2016).

## Results

Four dogs were enrolled between June 30, 2015 and August 8, 2016 (Table 1). Dog 1 was referred for inclusion in the trial after glioblastoma multiforme was diagnosed following surgical resection at the referring institution. The other three dogs were enrolled in the study based on MRI findings consistent with a rostrotentorial glioma (Bentley *et al*. 2013). At the time of craniectomy, all dogs were receiving prednisone at 0.3–1 mg kg^−1^ day^−1^ and anticonvulsants including phenobarbital (three dogs) and zonisamide (two dogs) with one dog being on both medications. Only dog 3 had any significant co‐morbidities with a non‐healing right tibial fracture and recent implant infection and medicated hypothyroidism.

**Table 1:** Basic patient information

Dogs enrolled	Breed	Weight (kg)	Age (years)	Sex	Neurologic signs at time of craniotomy	Laboratory/Imaging abnormalities
1	Labrador retriever	37.5	10.5	Male neutered	Generalized seizures	ALP 264 (11–131 U/L)
2	French bulldog	9.3	9	Female spayed	Generalized seizures, mild obtundation, mild postural reaction deficits	Neutrophils 12.96 × 10^3^ (2.9–12 × 10^3^) Band neutrophils 1.19 × 10^3^ (0–0.45 × 10^3^) Abdominal imaging not performed
3	American Staffordshire terrier	16.3	9	Female spayed	Generalized and focal seizures	WBCs 15.6 × 10^3^ (5.5–13.9 × 10^3^) Neutrophils 14.8 × 10^3^ (2.9–12 × 10^3^) ALP 178 (11–131 U/L) Cholesterol 318 (124–264 mg/dL)
4	Boxer	30	5	Male neutered	Generalized seizures, mild obtundation, circling, postural reaction deficits	ALP 1570 (11–131 U/L) ALT, (9–105 U/L) Cholesterol 318 (124–264 mg/dL)

ALP, Alkaline phosphatase; WBCs, white blood cells; ALT, alanine transferase.

All dogs’ objective contrast and non‐contrast enhancing pre and post‐ operative tumour volumes along with estimated tumour volumes and microcylinder numbers are summarized in Table 2. Tumour location MRI findings and histopathology results from all dogs are summarized in Table 3.

**Table 2:** Objective and estimated tumour volumes, microcylinder implantation #

Dogs	Pre‐operative tumour volume	Pre‐operative tumour volume	Immediate post‐operative tumour volume	Immediate post‐operative tumour volume	30 day post‐operative tumour volume	30 day post‐operative tumour volume	Estimated tumour volume (pre‐op max height x width x length)	# TMZ/GAD microcylinders implanted
1	6.08 cm^3^	6.36 cm^3^	0.13 cm^3^ (Volume at implantation)	0.2 cm^3^	0.41 cm^3^	0.41 cm^3^	8.9 cm^3^	3
2	1.53 cm^3^	1.86 cm^3^	1.47 cm^3^	1.61 cm^3^	Deceased	Deceased	4.7 cm^3^	12
3	2.88 cm^3^	2.88 cm^3^	2.76 cm^3^	2.86 cm^3^	0 cm^3^	0 cm^3^	6.5 cm^3^	6
4	1.34 cm^3^	6.86 cm^3^	0.78 cm^3^	7.38 cm^3^	4.32 cm^3^	5.03 cm^3^	10 cm^3^	5

**Table 3:** Tumour location, MRI findings and histopathology

Dogs	Mass location	Major MRI findings	Histopathology
1	Right frontal lobe	**Initial pre‐op:** mild transtentorial herniation **Pre implantation:** Peripheral contrast enhancement ventral right olfactory bulb (presumed residual tumor)	**Initial:** Glioblastoma multiforme **Implantation:** fibrous tissue with lymphocytic, neutrophilic inflammation
2	Right frontal lobe	**Pre implantation:** Marked T2W hyperintensity, meningeal contact rostrally, marked contrast enhancement **Post implantation:** Several microcylinders extend into normal brain parenchyma	Oligodendroglioma
3	Right frontal lobe	**Pre implantation:** Marked T2W hyperintensity, meningeal contact rostrally, mild contrast enhancement **Post implantation:** Mild pneumocephalus, microcylinder tract communicating with lateral ventricle	Oligodendroglioma (Grade II)
4	Right temporal lobe	**Pre implantation:** Marked T2W hyperintensity, meningeal contact laterally, marked contrast enhancement, mild haemorrhage within tumour, moderate transtentorial herniation	Oligodendroglioma (Grade II)

In all dogs the microcylinder placement was adequate for the amount of contrast‐enhancing tissue that was present (≥1 microcylinder per 1 cm^3^ suspected tumour). In dog 1, the microcylinders were placed more closely together (<0.5 cm) than desired. In dog 2, several of the microcylinders extended just past the tumour margin into brain parenchyma. Microcylinders implanted in columns were placed directly adjacent to each other to prevent lack of tumour coverage in that plane. In dog 3, one microcylinder and implantation tract could be seen extending to and abutting the most rostral aspect of the right lateral ventricle. A small blooming signal void in all sequences consistent with intraparenchymal air was present at the dorsocaudal aspect of the resection margin, but did not appear to abut the lateral ventricle and did not appear to communicate with the subarachnoid space.

All dogs recovered well from the implantation procedure. A fentanyl constant rate infusion (CRI) (2–5 mcg kg^−1^ h^−1^ IV) was used for the first 12–24 h following surgery in all dogs. Pain medication was either discontinued at that time (dog 1) or continued with buccal buprenorphine 0.01 mg kg^−1^ q6–8h (dog 2), tramadol 6.5 mg kg^−1^ PO q8h (dog 3) or tramadol 1.7 mg kg^−1^ PO q8h and gabapentin 10 mg kg^−1^ PO q8h (dog 4). Additionally, in dog 1 prednisone (0.27 mg^−1^ kg^−1^ PO q24h), phenobarbital (2.6 mg kg^−1^ PO q12h) and omeprazole (1 mg kg^−1^ PO q24h) were continued. For dog 2 previous doses of phenobarbital (3.4 mg kg^−1^ PO q12h) omeprazole (1.1 mg kg^−1^ PO q24h) and zonisamide (10.1 mg kg^−1^ PO q12h) were continued. Prednisone was administered (1 mg kg^−1^ PO q24h) and clindamycin was prescribed for 2 weeks post‐operatively at 15 mg kg^−1^ PO q12h. For dog 3 cephalexin was prescribed at 33 mg kg^−1^ PO q12h for 2 weeks post‐operatively due to the non‐healing wound associated with the previous tibial fracture. Prednisone (0.6 mg kg^−1^ PO q24h), phenobarbital (2 mg kg^−1^ PO q12h) and thyroxine (0.02 mg kg^−1^ PO q12h) were continued. The patient was discharged 5 days post‐operatively with those medications and trazodone (3 mg kg^−1^ PO q8–12h) as needed. In dog 4 cefazolin was continued at 22 mg kg^−1^ IV q8h for 2 days after surgery. He was discharged 3 days post‐operatively with the previous zonisamide and prednisone doses and the addition of gabapentin (10 mg kg^−1^ PO q8h) and trazodone (1.7 mg kg^−1^ PO q8h).

Dog 1 had a normal neurologic examination the morning following surgery, although he did appear more anxious than pre‐operatively. Dog 1 was discharged 3 days following surgery and had no identifiable post‐operative complications. Dog 2 had mild worsening of her neurologic deficits post‐operatively, which included circling to the right and worsened postural reaction deficits on her left side; both were improving by the time of discharge 72 h post‐operatively. Dog 3 developed no post‐operative deficits and was discharged by 72 h post‐operatively. Dog 4 developed mild right‐sided facial paresis and mild left‐sided postural reaction deficits after surgery, which resolved over the course of 3 days, when the dog was discharged.

Dog 1 underwent post‐surgical MRI using a 1.5T Toshiba Vantage Titan MRI at 30 days post‐implantation at the University of Florida veterinary teaching hospital (UF‐VTH). The appearance of the MR images at 30 days following surgery was very similar to the immediate post‐operative imaging, though the TMZ/Gd microcylinders were not clearly visible. At the time of follow‐up MRI Dog 1 had not had any seizures since implantation and had a normal neurologic exam.

**Figure 3:**
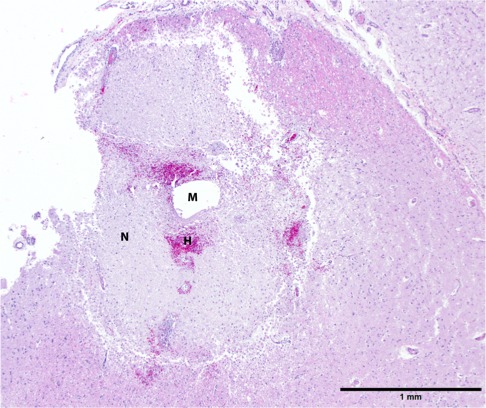
Peritumoral neuroparenchyma at implantation site. There is an empty spherical hole (M) interpreted to be a microcylinder tract that is surrounded by a circumferential rim of eosinophilic necrotic debris (N), about 1 mm in width, with multifocal hemorrhage (H) and a mild infiltration of gitter cells. Note the lack of significant inflammation in surrounding neuroparenchyma. 40x. HE staining.

Approximately 9 days after discharge, dog 2 suddenly began experiencing severe lethargy. She was presented to an emergency veterinarian and diagnosed with a hemoabdomen and suspicion for a splenic mass. The owner elected humane euthanasia. Necropsy results indicated that the cause of the hemoabdomen was splenic hemangiosarcoma without evidence of metastasis. The brain tumour was confirmed to be an oligodendroglioma and was traversed by numerous vessels lined by severely hyperplastic endothelium. Some intratumoral vessels were necrotic and surrounded by necrosis and hemorrhage. At the site of implantation, there were multiple empty microcylinder tracts that were surrounded by a rim of necrosis with hemorrhage and variable numbers of infiltrating gitter cells (Fig. 3). Adjacent vessels had minimal lymphocytic perivascular infiltrations.

At 1 month following craniectomy, dog 3 was presented for follow‐up MRI. She appeared neurologically normal and had not had any seizure activity since discharge. At that time, MRI showed no definitive tumour tissue and a static appearance to the post‐operative craniectomy site. However, moderate pneumocephalus was diagnosed in the right lateral ventricle, third ventricle and caudal aspect of the left lateral ventricle based on rounded areas of blooming signal void on all imaging series. The small area of intraparenchymal pneumocephalus from the immediate post‐implantation MRI was no longer apparent. Because the patient was asymptomatic and it was unclear whether her pneumocephalus was stable/resolving versus progressing at that time point, the decision was made to continue her prednisone, phenobarbital and thyroxine therapy and monitor for neurologic deterioration. MRI evaluation 3 months post‐implantation revealed progressive pneumocephalus, which was surgically addressed at that time, resulting in resolution on MRI performed 4 months post‐implantation.

At 1 month post‐craniectomy, dog 4 presented to the local specialty referral center for a follow‐up MRI. He was neurologically normal but had experienced three generalized seizures since discharge. At that time, MRI showed substantial tumour regrowth with marked herniation of tissue through the previous craniectomy site. Dog 4 was re‐evaluated at UGA 2 weeks later and had developed right‐sided circling, left‐sided postural reaction deficits and experienced further seizure activity. At that time, he was withdrawn from the study so that additional treatments could be performed.

Dog 1 had previously been diagnosed with a glioblastoma multiforme; however, histopathology of the surgical biopsies taken at the time of implantation showed only fibrous tissue with moderate to marked lymphocytic and neutrophilic inflammation and prominent blood vessels, consistent with surgical healing. Dog 2 had histopathology of the mass consistent with an oligodendroglioma. On histopathology for dog 3, olig2 immunopositivity of the mass indicated an oligodendroglioma, likely grade II given a moderate degree of cellular atypia with low to moderate mitotic activity. For dog 3, histopathology and olig2 immunopositivity confirmed an oligodendroglioma with vacuolar degeneration, likely grade II given mild anisocytosis and anisokaryosis.

## Discussion

Although local therapies for brain tumours are still in their infancy, there is significant theoretical promise in these types of treatments due to their ability to be delivered directly to tumour tissue rather than having to traverse the entire body and cross the blood‐brain‐barrier (BBB) in order to reach their target. While the BBB is likely disrupted in regions of abnormal tissue, such as surrounding tumours, systemic chemotherapy had not played a major role in the treatment of glioblastoma multiforme, the most common human malignant brain tumour (Louis *et al*. 2016), until oral temozolomide. With its capability to partially cross the BBB, it was found to extend the survival of human GBM patients compared to surgery and radiation therapy alone. Unfortunately, systemic chemotherapy must be administered at high enough doses to reach an effective concentration within the tumour tissue, which leads to significant adverse systemic effects in many people. In up to 15% of human GBM patients, oral TMZ must be discontinued, delayed or dose‐reduced due to toxicities. The combination of oral TMZ with local therapies, such as carmustine (Gliadel^®^) wafers, potentiates toxicity further (Miglierini *et al*. 2012). Alternatively, local delivery of TMZ via biopolymers has been shown to provide significantly longer survival and higher intracranial TMZ levels compared to oral administration in rodent glioma models with or without adjunctive radiation therapy (Brem *et al*. 2007).

Gliadel^®^ wafers have been the most promising and utilized local chemotherapy option developed to date. Their large size, however, only allows intra‐cavitary implantation into the resection bed rather than interstitial intratumoral, minimally invasive implantation. There is not published information on the distribution dynamics of carmustine within human glioma resection cavities, but tissue penetration is theorized to be only a few millimeters via diffusion (Fleming & Saltzman 2002). Because of their infiltrative nature, 80–95% of malignant gliomas recur within 2 cm of the resection cavity; Gliadel^**®**^ wafers, therefore, lead to a large “treatment gap” of the region of recurrence that could be overcome with intraparenchymal implants (Massey & Wallner 1990). Additionally, the use of Gliadel^®^ wafers placed into a resection cavity has been associated with cerebral edema, healing abnormalities of the surgical site, CSF leaks, intracranial infections, seizures, hydrocephalus and cyst formation (De Bonis *et al*. 2012).

Entotherapy is a novel cancer treatment utilizing drug‐eluting microcylinders that can be image‐guided and also identified via post‐implantation imaging. PLGA is an attractive delivery device for entotherapy due to its well‐established biocompatibility, lack of neurotoxicity, degradation by hydrolysis and lack of T‐lymphocyte response (Veziers *et al*. 2001; Voges *et al*. 2002; Fournier *et al*. 2003; Menei *et al*. 2005b). Additionally, the ratio of lactide to glycolide can be adjusted to allow varied rates of degradation (Veziers *et al*. 2001). For example, a ratio of 1:1 allows for a degradation time of approximately 30 days, which is what was used in the previous safety study; (Hicks *et al*. 2016) the ratio was altered to allow a 90‐day degradation time for this pilot study, as this would cover the time period for post‐operative radiation therapy in human and canine patients. We have already demonstrated that PLGA microcylinders conjugated with gadolinium are visible on post‐implantation MRI in dogs and have proposed neuro‐navigational guidance for minimally invasive implantation of tumours that are not surgically accessible or when a more invasive craniotomy is declined (Hicks *et al*. 2016). This gives us an excellent groundwork to make entotherapy a realistic treatment option in brain tumours.

TMZ is ideal as a locally delivered chemotherapeutic because it has proven efficacy against GBM. It does not require hepatic activation and it rapidly hydrolyzes in a normal or alkaline pH; in the acidic environment of a tumour, TMZ is taken up into cells where it is converted to its active metabolite (Menei *et al*. 2005c). Such properties allows for excellent therapeutic effects while being safe to normal brain tissue surrounding a tumour. Some evidence for improved outcome in local biopolymer‐delivered TMZ versus oral TMZ therapy has been achieved in rat glioma models. Rats receiving intracranial TMZ with or without radiation therapy survived significantly longer than oral therapy controls, with or without radiation therapy (Brem *et al*. 2007). Intratumoral TMZ has also been shown to attenuate the immune‐suppression caused by oral TMZ in murine glioma models, leading to improved survival (Fritzell *et al*. 2013).

In our current clinical trial, the goal was to combine subtotal tumour resection with entotherapy in order to reduce morbidity following craniectomy and lead to a positive treatment outcome. This model also allowed us to evaluate whether the microcylinders could be used effectively in the presence of gross disease, which would be necessary if they were implanted into unresectable tumours. With our initial trial patients, the primary goal was the feasibility of performing this procedure on typical, tumour‐bearing dogs and evaluating whether free‐hand placement could be performed with a successful short‐term outcome. Subtotal resection was performed in the majority of cases in order to reduce the risk of morbidity associated with more aggressive tumour resection, especially given that apparent gross total resection during surgery in dogs often appears to be incomplete on follow‐up imaging, as in the case of dog 1. Additionally, information in humans indicates that there is little survival benefit of increased tumour resection for malignant glioma unless true gross total resection is achieved (Smets *et al*. 2013). In dog 3, where definitive residual tumour was noted post‐operatively on MRI, no obvious tumour could be detected on the 30 day post‐implantation MRI. Unfortunately, due to an unrelated neoplasm, dog 2 was euthanized too soon after implantation to adequately judge any therapeutic effect. Microcylinders were not located within the neoplastic tissue on necropsy, but may not have been trimmed in to the reviewed slides or may have dissolved during processing, though that did not occur in our previous study in which the same processing was performed. Around microcylinder tracts found in adjacent tissue there was a small (1 mm) area of necrosis. Given that TMZ is not known to be active in a normal tissue environment, it is most likely that this necrosis was related to the physical trauma associated with implantation. However, if the adjacent tissue was abnormal due to its proximity to the neoplastic mass, it is possible that TMZ could have been activated.

Dog 4, who had the largest amount of initial maximum tumour volume in comparison to the number of microcylinders implanted, showed signs of regrowth on the 30 day post‐implantation MRI. Since the trial included a small number of dogs, it is not possible to determine whether regrowth was due to inadequate tumour coverage by TMZ, however, post‐operative MRI suggests this is possible in dog 4 (Fig. 2). This would be consistent with findings in rodent glioma models where an increased intracranial TMZ dose provided a significant survival benefit (Brem *et al*. 2007). An implantation number meeting or even exceeding one microcylinder per 1 cm^3^ original tumour size may be most appropriate in dogs that only undergo partial resection. As shown in dog 2, implantation that far exceeds coverage for the original tumour size appears to be well‐tolerated and may improve control by treating neoplastic cells within grossly normal brain. Given the relatively wide area of recurrence around the cavity of a grossly resected tumour in humans, implantation of TMZ microcylinders into surrounding brain tissue that appears normal is probably necessary and was tolerated in dog 2, which is encouraging.

Because dog 1 had already undergone tumour resection with some healing prior to study referral, it was more difficult to judge possible residual tumour. At 30 days post‐implantation, there was not an obvious reduction of suspected residual tumour, but there was also a lack of progression. This could have been due to several factors including that: this dog was diagnosed with a higher grade tumour (grade IV astrocytoma) than any of the other study participants; he was implanted with the fewest microcylinders conveying the smallest total dose of TMZ (3 mg); or the suspected residual tumour tissue on the pre‐implantation MRI was not actually representative of neoplasia, a common problem in post‐surgical imaging of tumours (Chow *et al*. 2015). The lack of progression on the 30‐day post‐implantation MRI in this dog may indicate that tumour regrowth was delayed by the microcylinders. However, it may have been prudent to use a larger number of microcylinders, especially given that up to twelve TMZ/Gd microcylinders have been implanted into the normal brains of dogs without any significant clinical effect (Hicks *et al*. 2016).

With the exception of dog 2 no dog had a large enough number of TMZ/Gd microcylinders implanted to treat their original tumour volume. Given that a large number of microcylinders were well‐tolerated in dog 2, that a higher intratumoral TMZ dose improves survival over a lower dose in rats (Brem *et al*. 2007), and that free‐hand implantation, when used, leads to variability in microcylinder placement versus targeted implantation, an over‐estimated number of microcylinders should be considered.

This case series is not intended to evaluate the intermediate and long‐term outcomes of these patients. Results were only reported to 30 days post‐implantation, as we would have expected adverse events to be most common immediately following microcylinder implantation and in the initial period of brain exposure to TMZ/Gd. Instead of long‐term outcome, the focus here is the feasibility and tolerability of free‐hand implantation of TMZ/Gd microcylinders into partially resected rostrotentorial gliomas in dogs. We found that free‐hand implantation is feasible for surgically accessible gliomas and that there appears to be low morbidity associated with the combination of craniectomy and implantation. This would allow TMZ/Gd microcylinders to be utilized by practitioners that do not have access to stereotactic navigation, and is the most realistic type of implantation possible following surgical resection, as neuronavigation instrumentation that accounts for resected tumour is not yet commonly utilized. However, if free‐hand implantation is performed instead of using stereotactic guidance, the risk for pneumocephalus, if a communicating tract between the ventricle and craniectomy site is formed, should be acknowledged. Even with pre‐implantation MRI and planning for trajectory based on visible anatomy, it is very difficult to exactly space TMZ/Gd microcylinders as desired without navigation, and given results thus far, an over‐estimation of the number of microcylinders needed per tumour is recommended along with closer estimated spacing of microcylinders to help ensure adequate coverage. Additionally, even if tumours are grossly resected, implantation of TMZ microcylinders into normal appearing surrounding brain may help in reducing local recurrence.

There are limitations to this study that, without their presence, would have allowed the data to be more robust. These include that decisions made regarding alteration of the protocol when free‐hand implantation had to be used instead of neuronavigation did not allow for a unified application of the procedure. Instead of being able to strictly adhere to exact tumour measurements and strict implantation guidelines, estimation of initial tumour volume that permitted a “safety factor” for microcylinder number was performed. This allowed us to set a maximum number of microcylinders that should be needed to get appropriate tumour coverage. However, the additional variable of allowing the surgeon to subjectively decide the amount of tumour resection and base the final implantation number on the impression of the amount of residual tumour tissue and its shape lead to significant differences in the actual number of microcylinders implanted per dog considering their true residual tumour volumes. Additionally, although this is likely a minor point as microcylinders could be located with a variety of sequences, a strict pre and post‐operative imaging protocol would enable exact comparisons where there might be sequence variability. Finally, as with any new treatment, the standardization of evaluating adverse events is an important aspect of judging safety. We felt confident given our previous study and the very low total dose of TMZ that systemic adverse events would not be seen. However, a standardized adverse events evaluation would have been beneficial rather than typical clinical evaluation performed in our patients.

In the future, the goal would be to apply this treatment for surgically inaccessible gliomas using stereotactic navigation. To date, neuronavigation has been used in one patient and efforts to refine the process and make it consistently reproducible in our patients are underway. Ultimately, a controlled, randomized trial evaluating dogs receiving craniotomy and resection with or without microcylinders will be necessary along with maximizing microcylinder dose for efficacy; this will require many patients and multiple centres to complete. Going forward procedures that are performed with free‐hand implantation will utilize an over‐estimation of TMZ/Gd microcylinders compared to initial tumour size to reduce the risk of under‐dosing and allow the best chance to evaluate the efficacy of this therapy. If treatment is consistently tolerated in clinical patients, adjunctive radiation therapy may provide an even greater benefit, as local TMZ may improve the efficacy of radiation therapy as well (Brem *et al*. 2007). This report serves to demonstrate the feasibility and tolerability of entotherapy in spontaneous canine gliomas and represents the first clinical use of this novel therapy. A more standardized protocol utilizing an over‐estimated microcylinder number for each patient and placement of TMZ microcylinders into local normal brain tissue will be needed in order to best assess long term efficacy of this therapy in dogs.

## Source of funding

Funding was provided by Microspherix LLC, Boca Raton, FL. Microspherix LLC created the concept of therapy that underlies this clinical trial.

## Conflict of interest

Microspherix LLC was founded by and is operated by Dr. Edward Kaplan, who appears as an author on this paper and initially conceived of this therapy. Dr. Kaplan participated in the study design and monitored implantation in at least one patient but had no direct care of patients and was not permitted to make treatment decisions or perform evaluations for any patients.

## Ethical statement

The authors confirm that the ethical policies of *Veterinary Medicine and Science*, as noted on the journal's author guidelines page, have been adhered to and the approval of the University of Georgia Institutional Animal Care and Use Committee (IACUC) has been received. No laboratory animals were used in the creation of these data.

## Contributions

JH participated in study design, managed multiple clinical patients and wrote the majority of the manuscript. SP participated in study design, performed craniectomies and implantation procedures, supervised management of clinical patients, participated in preparation of the manuscript and figures. GS managed multiple clinical patients and participated in preparation of the manuscript. CS participated in management of one of the clinical patients and participated in preparation of the manuscript. SH performed and interpreted MRI studies for all clinical patients, consulted on study design regarding imaging and prepared the imaging portions of the manuscript. EH interpreted all pathology samples and prepared the pathology portions of the manuscript. MK participated in study design, supervision of clinical patient management and preparation of the manuscript. JK fabricated the PLGA implants, performed volumetric analysis and participated in preparation of the manuscript in regards to volumetric analysis. EK conceived of the novel technique, participated in study design and preparation of the manuscript.
